# High-density lipoprotein inhibits ox-LDL-induced adipokine secretion by upregulating SR-BI expression and suppressing ER Stress pathway

**DOI:** 10.1038/srep30889

**Published:** 2016-07-29

**Authors:** Guohua Song, Xia Wu, Pu Zhang, Yang Yu, Mingfeng Yang, Peng Jiao, Ni Wang, Haiming Song, You Wu, Xiangjian Zhang, Huaxia Liu, Shucun Qin

**Affiliations:** 1 Institute of Atherosclerosis, Key Laboratory of Atherosclerosis in Universities of Shandong, TaiShan Medical University, Taian, China; 2 Institute of Nursing, TaiShan Medical University, Taian, China; 3 Central Hospital of Taian City, Taian, China; 4 Maternal and child health hospital of Daiyue District, Taian, China; 5 Hebei Collaborative Innovation Center for Cardio-cerebrovascular Disease and Hebei Key Laboratory of Vascular Homeostasis, Shijiazhuang, 050000, China

## Abstract

Endoplasmic reticulum stress (ERS) in adipocytes can modulate adipokines secretion. The aim of this study was to explore the protective effect of high-density lipoprotein (HDL) on oxidized low-density lipoprotein (ox-LDL)-induced ERS-C/EBP homologous protein (CHOP) pathway-mediated adipokine secretion. Our results showed that serum adipokines, including visfatin, resistin and TNF-α, correlated inversely with serum HDL cholesterol level in patients with abdominal obesity. *In vitro*, like ERS inhibitor 4-phenylbutyric acid (PBA), HDL inhibited ox-LDL- or tunicamycin (TM, an ERS inducer)-induced increase in visfatin and resistin secretion. Moreover, HDL inhibited ox-LDL-induced free cholesterol (FC) accumulation in whole cell lysate and in the endoplasmic reticulum. Additionally, like PBA, HDL inhibited ox-LDL- or TM-induced activation of ERS response as assessed by the decreased phosphorylation of protein kinase-like ER kinase and eukaryotic translation initiation factor 2α and reduced nuclear translocation of activating transcription factor 6 as well as the downregulation of Bip and CHOP. Furthermore, HDL increased scavenger receptor class B type I (SR-BI) expression and SR-BI siRNA treatment abolished the inhibitory effects of HDL on ox-LDL-induced FC accumulation and CHOP upregulation. These data indicate that HDL may suppress ox-LDL-induced FC accumulation in adipocytes through upregulation of SR-BI, subsequently preventing ox-LDL-induced ER stress-CHOP pathway-mediated adipocyte inflammation.

Since the discovery of the adipocyte as a ‘metabolically active organ’ which secretes hormones, cytokines and chemokines, there is growing interest in understanding its functions in obesity and weight loss^1^. Adipose tissue secretes several bioactive molecules called adipokines, such as tumor necrosis factor-α(TNF-α), interleukin-6 (IL-6) and monocyte chemoattractant protein (MCP-1)^2^. Visfatin and resistin represents novel adipokines of the visceral adipose tissue^3^,^4^. Chronic low-grade inflammation is identified as a hallmark in obesity with dysregulation of adipose tissue-derived adipokines production. These are detected in both serum and adipose tissue and are active factors that modulate the effects of obesity and related comorbidities^5^,^6^.

Newly synthesized secretory and membrane-associated proteins are correctly folded and assembled by chaperones in the endoplasmic reticulum (ER)^7^. Failure of the ER’s adaptive capacity results in activation of the ER stress, also known as unfolded protein response (UPR). It is known that ER stress is increased in liver and adipose tissue of obese mice^8^,^9^ and obese human subjects^10^. Induction of ER stress in fat tissues modifies adipokines secretion and induces inflammation^11^. Recently studies have demonstrated that oxidized low-density lipoprotein (ox-LDL) can promote the expression and secretion of visfatin and resistin by activation of ER stress-C/EBP homologous protein (CHOP) pathway, which may be related to the increase of cholesterol load in adipocytes, whereas tauroursodeoxycholic acid (TUDCA) protects adipocytes from ox-LDL-induced adipokine secretion by inhibiting ERS activation^12^. So it could be presumed that inhibition of ER stress may be an effective approach to modify adipokines secretion, inhibit inflammation and reduce the risk of obesity and its complications.

It is well-known that high-density lipoprotein (HDL) has significant anti-inflammatory and anti-atherogenic properties. However, the mechanism by which HDL prevents atherogenesis has not been completely elucidated. Recent studies have implicated a strong link between circulating plasma resistin and atherosclerosis^13^. Further, HDL may diminish lipid accumulation in adipocytes^14^, which could possibly account for the favourable effects of HDL on adipokines secretion. Therefore, we hypothesized that regulation of adipokines secretion and lipid accumulation in adipocytes by HDL might contribute to its anti-atherogenic properties. In the present study, we investigated the effect of HDL on adipokines secretion and lipid accumulation, with emphasis on its role in downregulating the ER stress-CHOP pathway-mediated inflammation *in vitro* and *in vivo*.

## Results

### Association of serum HDL cholesterol and adipokines levels in subjects with abdominal obesity

**Figure 1:**
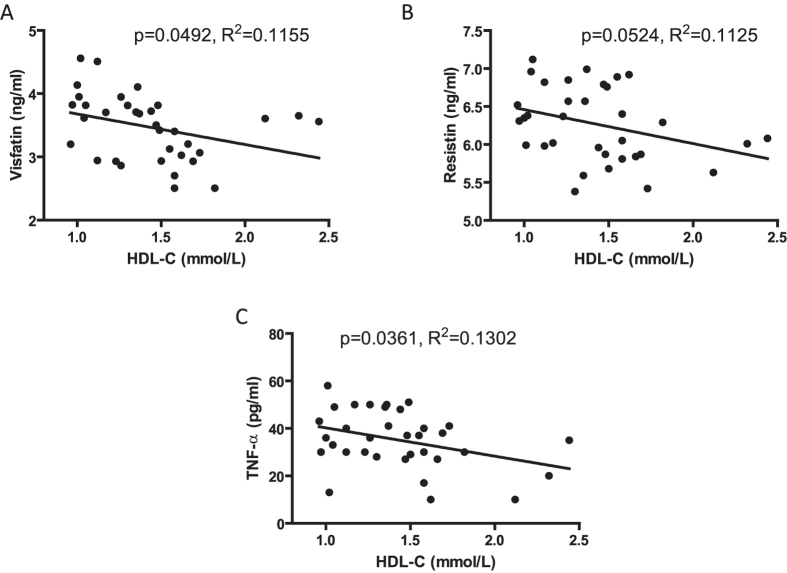
Association of serum HDL cholesterol and adipokines levels in subjects with abdominal obesity. 34 subjects with abdominal obesity were enrolled. (**A**) Scatter plot of HDL-C (mmol/L) and visfatin (ng/ml) with linear regression analysis. (**B**) Scatter plot of HDL-C (mmol/L) and resistin (ng/ml) with linear regression analysis. (**C**) Scatter plot of HDL-C (mmol/L) and TNF-α (ng/ml) with linear regression analysis.

The baseline demographics of subjects are presented in Supplemental Table 1. Thirty-four subjects with abdominal obesity were enrolled in the study and no smokers or drinkers were included. As shown in Fig. 1A, a negative correlation was noted between serum visfatin level and serum HDL-C. The same correlation was found between HDL-C with resistin and TNF-α (Fig. 1B,C).

### HDL attenuates ox-LDL-induced secretion of visfatin and resistin in 3T3-L1 differentiated adipocytes

**Figure 2:**
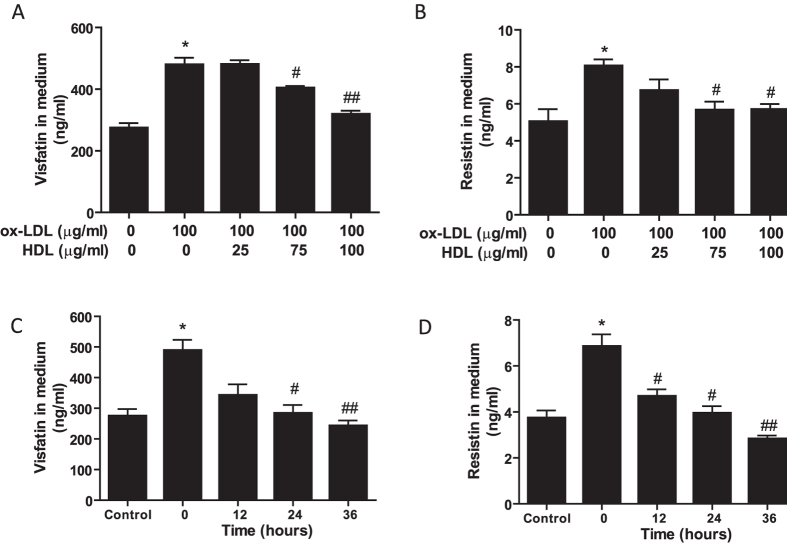
HDL attenuates ox-LDL-induced secretion of visfatin and resistin in 3T3-L1 differentiated adipocytes. (**A**,**B**) Adipocytes were pretreated with different concentrations of HDL in DMEM with 1% FBS for 2 hr, followed by incubation with ox-LDL and 10 μg/ml Sandoz 58035 for 48 hrs, and then visfatin and resistin levels in cell medium were determined by Elisa assay. (**C**,**D**) Adipocytes were incubated with ox-LDL and sandoz58035 (10 μg/ml) for 48 hr in a combination with HDL (100 μg/ml) for various times, and then amounts of visfatin and resistin released to cell medium were determined by Elisa assay. Data are presented as the mean ± SEM of at least six independent experiments. *P < 0.05, **P < 0.01 versus control group; ^#^*P *< 0.05, ^##^*P *< 0.01 versus ox-LDL or 0 hr group.

Based on the negative correlation of HDL-C and pro-inflammatory adipokines in patients with obesity, an *in vitro* ox-LDL-induced inflammatory adipocyte model was established to investigate the protective function of HDL. As shown in Fig. 2, incubation of adipocytes with ox-LDL led to significant upregulation of visfatin and resistin release, whereas the upregulation was markedly attenuated by pretreatment with HDL in a dose-dependent (Fig. 2A,B) and time-dependent manner (Fig. 2C,D).

### HDL inhibits ox-LDL-induced free cholesterol accumulation in whole adipocytes and in ER-enriched membranes

**Figure 3:**
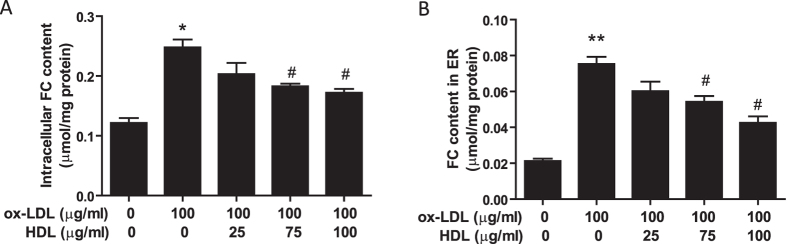
HDL inhibits ox-LDL-induced free cholesterol accumulation in whole adipocytes and in ER-enriched membranes. Adipocytes were pretreated with HDL in DMEM with 1% FBS for 2 hr, followed by incubation with ox-LDL and 10 μg/ml Sandoz 58035 for 48 hr, and then (**A**) FC content in the whole cells was analyzed by a commercially available kit. (**B**) ER-enriched membranes from adipocytes were fractionated by sucrose step gradient centrifugation as described under the “Methods”, and the pellet fraction was assayed for FC content. Data are presented as the mean ± SEM of at least four independent experiments. *P < 0.05, **P < 0.01 versus control group; ^#^*P *< 0.05 versus ox-LDL group.

We examined endocytic uptake of FC using the differentiated 3T3-L1 cell line. Ox-LDL is a rich source of FC. ACAT inhibitor Sandoz 58035 was used to inhibit FC transforming into cholesterol ester in order to enhance cellular FC concentration. As shown in Fig. 3A, ox-LDL significantly increased intracellular cholesterol concentration while pretreatment with HDL inhibited FC accumulation in adipocytes. Further, we isolated the ER-enriched fraction in adipocytes and as shown in Fig. 3B, ox-LDL increased the FC content in the ER-enriched fraction, which was prevented by HDL pretreatment in a dose-dependent manner.

### HDL suppresses secretion of visfatin and resistin induced by ox-LDL or TM in 3T3-L1 adipocytes

**Figure 4:**
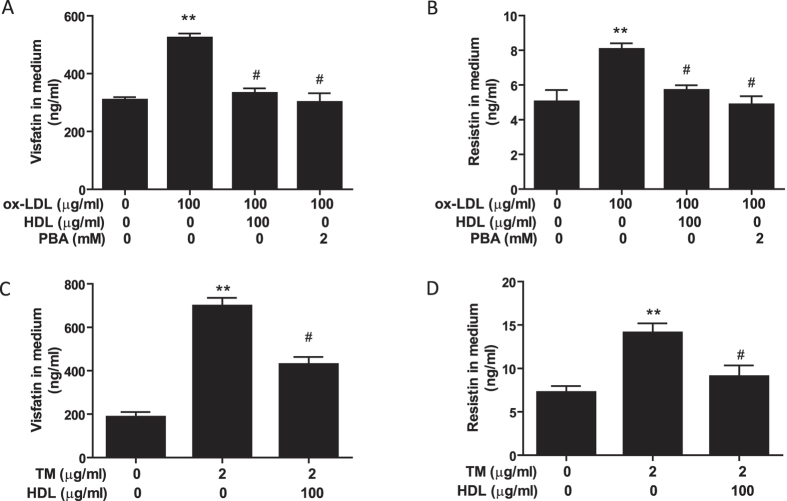
HDL suppresses secretion of visfatin and resistin induced by ox-LDL or TM in 3T3-L1 adipocytes. (**A**,**B**) Adipocytes were pretreated with HDL or PBA in DMEM with 1% FBS for 2 hr, followed by incubation with ox-LDL and sandoz58035 (10 μg/ml) for 48 hr. (**C**,**D**) Adipocytes were pretreated with HDL or PBA in DMEM with 1% FBS for 2 hr, followed by incubation with TM and sandoz58035 (10 μg/ml) for 24 hr. Visfatin and resistin levels in cell medium were determined by Elisa assay. Data are expressed as the mean ± SEM of at least 5 independent experiments. **P < 0.01 versus control group; ^#^*P* < 0.05 versus ox-LDL or TM group.

Next, we investigated the relative contribution of ER stress-mediated adipocyte inflammation to the protective effect of HDL. PBA, an ER stress inhibitor, and TM, an ER stress inducer, were used to inhibit and induce ER stress, respectively. As shown in Fig. 4A,B, similar to PBA, HDL blocked the increased visfatin and resistin secretion induced by ox-LDL. Conversely, similar to ox-LDL, TM increased adipokines secretion, which was also prevented by HDL pretreatment (Fig. 4C,D). These findings indicate that HDL can reduce ER stress, which may contribute to its inhibitory effect on ox-LDL-induced adipocyte inflammation.

### HDL mitigates ER stress response in 3T3-L1 adipocytes induced by ox-LDL or TM

**Figure 5:**
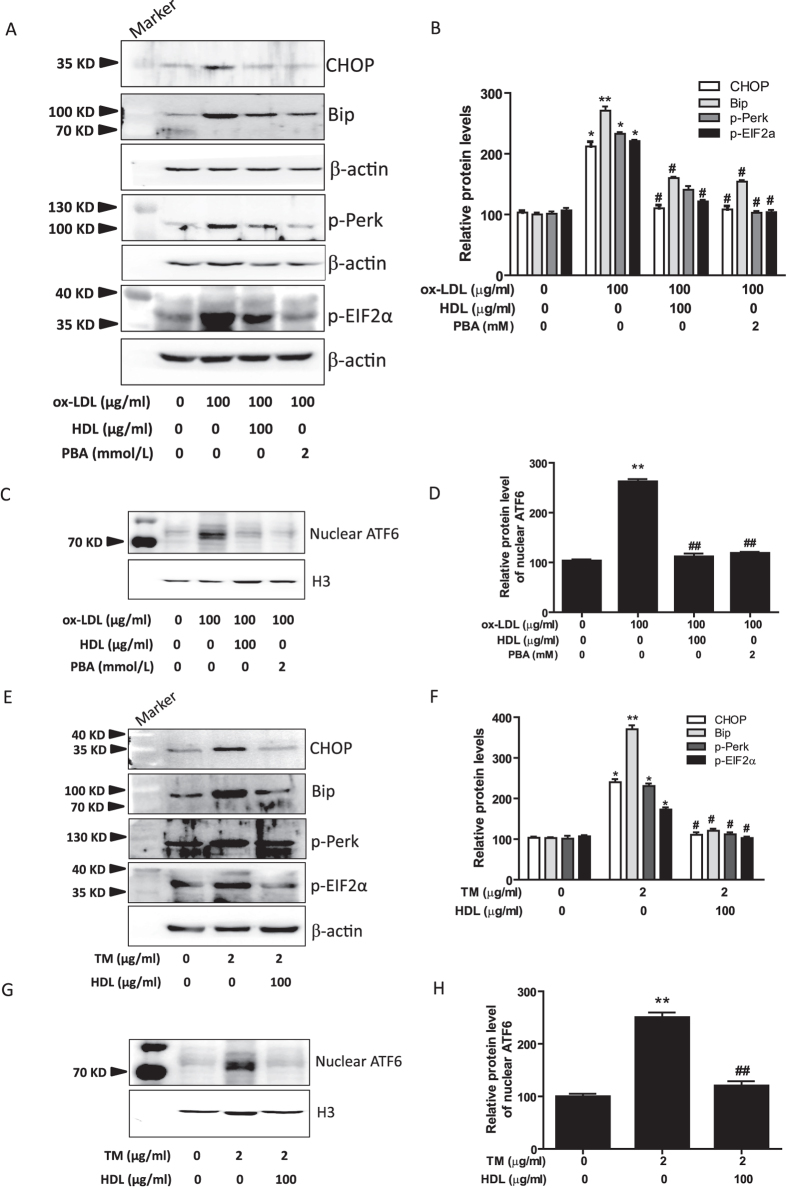
HDL suppresses ER stress response in 3T3-L1 adipocytes induced by ox-LDL or TM. (**A**–**D**) Adipocytes were pretreated with HDL or PBA in DMEM with 1% FBS for 2 hr, followed by incubation with ox-LDL and Sandoz58035 (10 μg/ml) for 48 hr, and then (**A,B**) the protein levels of ER stress markers were evaluated by Western blot. (**C**,**D**) The levels of ATF6 protein in nuclear were analyzed by Western blot and normalized to histone H3 level. (**E–H**) Adipocytes were pretreated with or without HDL for 2 hr, and then followed by incubation with TM and sandoz58035 for 24 hr, and then protein levels of ER stress markers and nuclear ATF6 were determined by Western blot. Data are expressed as the mean ± SEM of at least four independent experiments. *P < 0.05, **P < 0.01 versus control group; ^#^*P* < 0.05, ^##^*P* < 0.01 versus ox-LDL or TM group.

To further confirm the involvement of ERS in the anti-inflammatory effects of HDL on adipocytes, we evaluated the changes in CHOP and its two important upstream molecules, protein kinase-like ER kinase (PERK) and activating transcription factor 6 (ATF6). As shown in Fig. 5A–D, ox-LDL increased the activation of ER stress-CHOP pathway as assessed by phosphorylation of PERK and eukaryotic translation initiation factor 2α (eIF2α), and nuclear translocation of ATF6, with concomitant upregulation of Bip and CHOP. Moreover, pretreatment with HDL and PBA significantly inhibited the ox-LDL-induced ER stress response. Nextly, we investigated the regulatory effects of HDL on TM-induced ER stress response in adipocytes. As shown in Fig. 5E–H, pretreatment with HDL inhibited TM-induced activation of ER stress-CHOP pathway as determined by the reduced phosphorylation of PERK and eIF2α, and decreased ATF6 nuclear translocation and the expression of Bip and CHOP.

### HDL increases scavenger receptor class B type I (SR-BI) expression and inhibits ox-LDL-induced FC accumulation through SR-BI receptor in 3T3-L1 adipocytes

**Figure 6:**
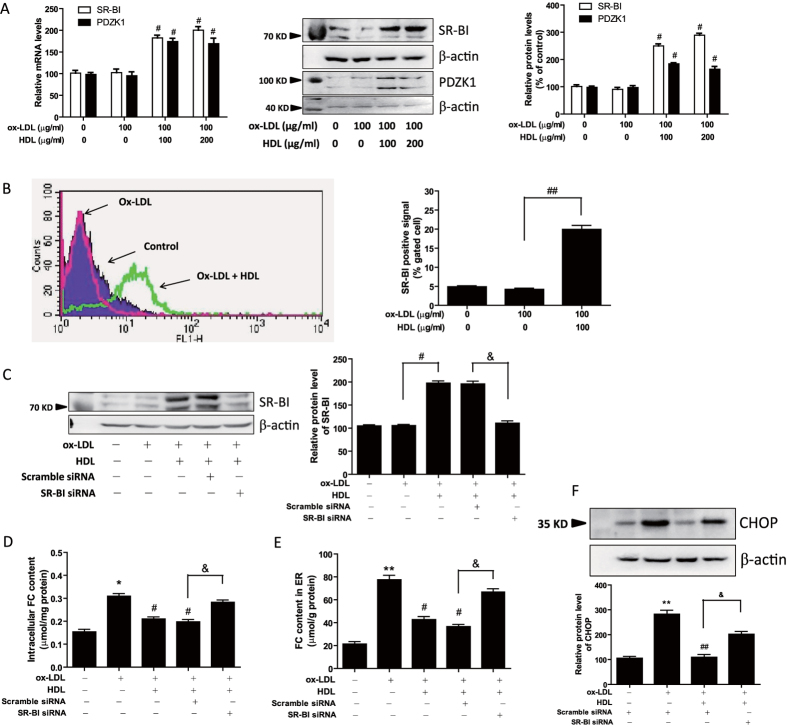
HDL increases SR-BI expression and inhibits ox-LDL-induced FC accumulation through SR-BI receptor in 3T3-L1 adipocytes. (**A**,**B**) Adipocytes were pretreated with HDL in DMEM with 1% FBS for 2 hr, followed by incubation with ox-LDL and sandoz58035 (10 μg/ml) for 48 hr, and then (**A**) the mRNA and protein levels of SR-BI and PDZK1 were evaluated by Real-time PCR and Western blot, and (**B**) the membrane expression of SR-BI was measured by flow cytometry. (**C**–**F**) Adipocytes were transfected with a siRNA against SR-BI or a negative scramble control siRNA for 48 hr, and then the cells were pretreated with HDL (100 μg/ml) for 2 hr, and followed by incubation with ox-LDL (100 μg/ml) and sandoz58035 (10 μg/ml) for 48 hr. After that, the silencing of SR-BI was validated by Western blotting (**C**). And FC levels in adipocytes (**D**) and ER-riched fractions (**E**) were analyzed by a commercially available kit. Further, the protein levels of CHOP (**F**), the ER stress marker, were evaluated by Western blot. Data are expressed as the mean ± SEM of at least four independent experiments. *P < 0.05, **P < 0.01 versus control group; ^#^*P* < 0.05, ^##^*P* < 0.01 versus ox-LDL group; ^&^*P* < 0.05 versus ox-LDL and HDL group transfected with scramble siRNA.

It has been reported that SR-BI plays an important role in promoting cholesterol transfer to HDL from adipocytes^15^. Further, we found HDL attenuated ox-LDL-induced free cholesterol accumulation in adipocytes (Fig. 3). Therefore, we next explored whether the mechanism underlying the inhibitory effect of HDL on ER stress-CHOP pathway could be through modulation of SR-BI expression and SR-BI-mediated cholesterol efflux. As shown in Fig. 6A, SR-BI expression was remarkably increased by HDL pretreatment in both mRNA and protein levels. Also, Flow cytometry data showed that membrane expression of SR-BI was increased by HDL pretreatment (Fig. 6B). It is known that SR-BI mediates several features of HDL metabolism and function, some of which depend on SR-BI’s interaction with PDZ domain containing 1 (PDZK1). Therefore, we determined the expression of PDZK1 and as shown in Fig. 6A, PDZK1 expression was increased by HDL pretreatment in both mRNA and protein levels.

**Figure 7:**
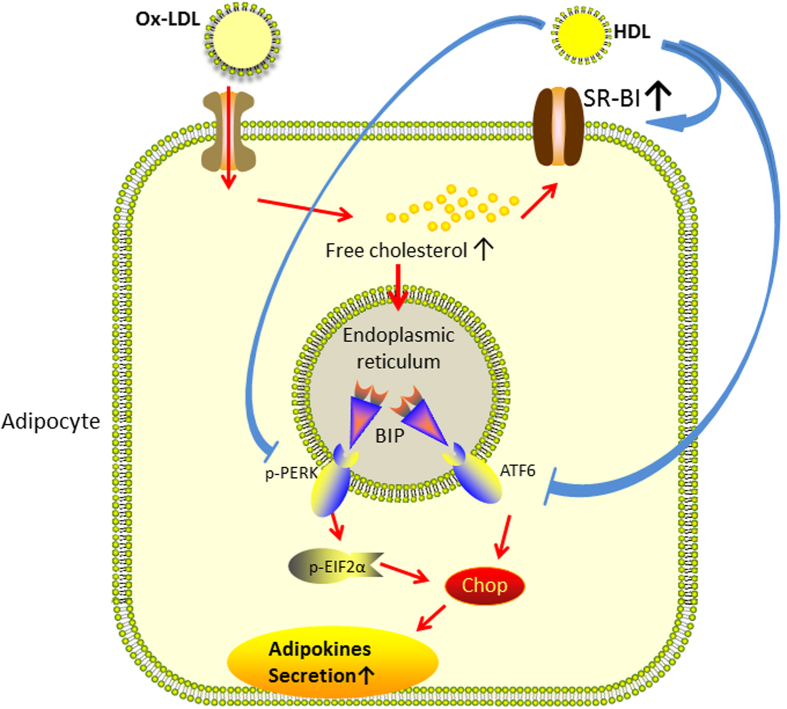
A schematic diagram illustrating that ox-LDL stimulated the expression and secretion of adipokines in adipocytes through activation of ER stress. HDL may suppress ox-LDL-induced FC accumulation in adipocytes through upregulation of SR-BI, subsequently preventing ox-LDL-induced ER stress-CHOP pathway-mediated adipocyte inflammation.

Moreover, we knocked down the expression of SR-BI by small interfering RNA (siRNA) and the efficiency of RNA interference was investigated by western blotting. As shown in Fig. 6C, SR-BI expression was inhibited by SR-BI siRNA in the presence of ox-LDL and HDL. The role of SR-BI on the inhibitory effects of HDL on ox-LDL-induced FC accumulation and CHOP upregulation was further determined. Results in Fig. 6D–F showed that SR-BI siRNA treatment abolished the inhibitory effects of HDL on ox-LDL-induced FC accumulation in whole cell lysates and ER-enriched fraction, and ox-LDL-induced CHOP upregulation. These data indicate that HDL may suppress ox-LDL-induced FC accumulation in adipocytes through upregulation of SR-BI, subsequently preventing ox-LDL-induced ER stress-CHOP pathway-mediated adipocyte inflammation. A schematic diagram is shown in Fig. 7.

## Discussion

The disruptions of cellular or organismal cholesterol homeostasis may lead to an augmentation of inflammatory responses. Chronic inflammation constitutes an important link between obesity and its pathophysiological sequelae, including atherosclerosis, insulin resistance, or cancer development^16^. HDL can protect against the development of atherosclerotic coronary heart disease partly by promoting the efflux of cholesterol from macrophages in the artery wall. Besides, it can exert anti-inflammatory function in endothelial cells and macrophages^17^. In the present study, we demonstrate that HDL may suppress ox-LDL-induced FC accumulation in adipocytes through upregulation of SR-BI, subsequently preventing ox-LDL-induced ER stress-CHOP pathway-mediated adipocyte inflammation. For the first time, we confirms the protective effects of HDL on adipocyte inflammation, which provides the important link between obesity and its complications. Also, our finding is likely to provide a clue that therapeutic interventions such as increasing production or infusion, or improving the function of HDL may sever the links between cholesterol accumulation and inflammation in adipose tissues, and have beneficial effects in patients with obesity and metabolic syndrome.

It has been reported that adipose tissue is not only an energy-regulating organ, but also an important endocrine organ, which can secrete various adipokines and chemokines. Interestingly, it has been demonstrated that adipocytes have the potential to be very efficiently and rapidly converted into macrophages and robustly secrete cytokines and chemokines^18^, suggesting that adipocytes may play a potential role in the inflammatory process of obesity-associated diseases. Visfatin and resistin represents novel pro-inflammatory adipokines of the visceral adipose tissue^4^,^19^. Increasing evidence strongly suggests that ox-LDL causes inflammation in different cell types, such as macrophages^20^ and adipocytes^21^. ox-LDL is taken up by CD36 on adipocytes and results in a large accumulation of cholesterol in cells, which results in disruption of lipid homeostasis. Furthermore, cellular accumulation of excess cholesterol may serve as a trigger for inflammation, which might contribute to obesity-induced insulin resistance and diabetes^22^. We have reported that HDL can protect macrophages from ox-LDL-induced inflammation^23^, however, whether HDL could protect adipocytes against ox-LDL-induced inflammation has not been studied. Our data demonstrated that differentiated adipocytes treated with ox-LDL accumulated significant amounts of free cholesterol in whole cells and in ER, with a concomitant increase in secretion of visfatin and resistin. In contrast, addition of HDL to ox-LDL-treated adipocytes reduced the FC accumulation and decreased the adipokines secretion in a dose-dependent and time-dependent manner.

Clinical evidence as well as experimental results strongly suggests the major contribution of ER stress to obesity-induced insulin resistance and diabetes^24^,^25^,^26^. ER stress activation can markedly induce the secretion of inflammatory cytokines, such as IL-6, in adipocytes^27^. Further, the recent study has demonstrated that ox-LDL promoted the expression and secretion of visfatin and resistin through its activation of ER stress in 3T3-L1 adipocytes^12^. Previously, we have observed that HDL reduce the expression and secretion of TNF-α and IL-6 in macrophages^23^,^28^, suggesting that HDL has direct anti-inflammatory effects on macrophages. Other reports have shown that HDL inhibits ER stress response induced by ox-LDL in human endothelial cells^29^ and in macrophages^30^. However it is still unclear whether HDL has inhibitory effect on ER stress of adipocytes. In the present study, we explored the impact of HDL on ox-LDL-induced ER stress and proinflammatory adipokines release in 3T3-L1 adipocytes. We found for the first time that similar to chemical chaperone PBA, HDL pretreatment significantly suppressed the ox-LDL-induced expressions of CHOP by inhibiting nuclear translocation of ATF6 and phosphorylation of PERK and eIF2α. Also, we found HDL inhibited ox-LDL-induced secretion of visfatin and resistin in adipocytes. TM has been known to induce inflammation by activating ER stress signaling pathways. Therefore, we further examined the effect of HDL on TM-induced inflammation in adipocytes. We found that HDL attenuated TM-induced secretion of adipokines and upregulation of CHOP and Bip expression. The activation of ATF6 and phosphorylation of PERK and eIF2α induced by TM was also significantly suppressed by HDL. The results suggest that HDL exerts anti-inflammatory actions in adipocytes at least partly through inhibition of ER stress and HDL may suppress the activation of the two crucial upstream signals, nuclear translocation of ATF6 and phosphorylation of PERK, thereby inhibiting the ER stress-CHOP pathway. Moreover, amelioration of ER stress in adipocytes may be another mechanism of the pleiotropic actions of HDL. These findings enrich our understanding of the beneficial effects of HDL.

Next, we need to know the mechanism by which HDL affect the upstream signals of the ER stress-CHOP pathway. It has revealed that ER has very low cholesterol content, the accumulation of FC in this cellular organelle may induce membrane dysfunction and subsequent ER stress^31^. The majority of cholesterol in adipocytes originates from circulating lipoproteins, as a consequence of the low activity in cholesterol de novo synthetic pathway^32^. It has been shown that adipocytes have the potential to be very efficiently and rapidly converted into macrophages to uptake and degrade ox-LDL^18^. Previous report from Zhao SP has demonstrated that adipocytes can endocytic uptake and degrade cholesterol from ox-LDL^33^. In the present study, we found HDL inhibited the ox-LDL-induced cholesterol load in whole adipocytes and in the ER. Therefore it could be possible that HDL might inhibit the activation of ER stress by decreasing the cholesterol load in adipocytes and ER. Further, it is known that adipocytes can transfer cholesterol to HDL *in vivo* as well as *in vitro*, which is dependent on SR-BI, but not ABCG1, in adipocyte cholesterol efflux to mature HDL. In our study, we found that HDL treatment markedly increased SR-BI expression and SR-BI knocking down abolished the inhibitory effects of HDL on ox-LDL-induced FC accumulation in whole cell lysates and ER-enriched fraction, and ox-LDL-induced CHOP upregulation. These data indicate that HDL may suppress ox-LDL-induced FC accumulation in adipocytes through upregulation of SR-BI, subsequently preventing ox-LDL-induced ER stress-CHOP pathway-mediated adipocyte inflammation. Although it needs further study, this provides a possible mechanism for the inhibition of adipocytes ER stress by HDL. Otherwise, ox-LDL is a marker of lipoprotein-associated oxidative stress. Oxidative stress is a common insult that can lead to ER stress. HDL is known to suppress oxidative stress, so HDL may inhibit ER stress through its anti-oxidative stress properties. Other constituents and biological properties of HDL may also have effect on ER stress and SR-BI expression, therefore, we tested the content of bacterial endotoxin, which present in HDL particles. The data shows that the endotoxin content in HDL particles was 0.023 ± 0.015 EU/100 μg HDL protein (Supplemental Table 1). This concentration is very low and not sufficient to induce the upregulation of genes in adipocytes. In addition, microRNAs are known to be transported by HDL and the correlation of miRNAs, adipokines secretion and SR-BI expression needs to be further elucidated.

In conclusion, the present study confirmed the negative relation of serum HDL cholesterol and pro-inflammatory adipokines levels in subjects with abdominal obesity. We indicated that ox-LDL stimulated the expression and secretion of visfatin and resistin in 3T3-L1 adipocytes through activation of ER stress. HDL may suppress ox-LDL-induced FC accumulation in adipocytes through upregulation of SR-BI, subsequently preventing ox-LDL-induced ER stress-CHOP pathway-mediated adipocyte inflammation. These finding are likely to further enrich the pathophysiology of adipocytes and explain the favorable effect of HDL on obesity-related inflammatory diseases like diabetes or atherosclerosis.

## Methods

### Subjects

All experimental procedures were approved and carried out in accordance with the guidelines of the Ethics Committee of TaiShan Medical University. This cross-sectional study included selected 34 subjects who had abdominal obesity among adults participating in health check-ups at Maternal and child health hospital of Daiyue District. All participants provided written informed consent to participate before enrollment in the study.

Abdominal obesity (high triglycerides plus waist circumference [HTGWC]) was defined as triglycerides ≥1.7 mmol/L and waist circumference ≥90 cm (men) and ≥80 cm (women). We excluded patients with cardiovascular disease, diabetes mellitus, or lipid-lowering agents within 6 months before enrollment.

### Serum Analysis

Blood samples were obtained in the morning after an overnight fast. Serum HDL-C, LDL-C, triglycerides (TG), and glucose were measured by enzymatic methods on a chemical autoanalyzer (Hitachi Co 7600, Tokyo, Japan). Serum visfatin, resistin and TNF-α levels were measured by using the commercially available Elisa kit (Bluegene, Shanghai, China).

### Cell culture

3T3-L1 preadipocytes purchased from the American Type Culture Collection (ATCC) were maintained in DMEM (GIBCO) supplemented with 10% (v/v) fetal bovine serum (FBS, Gibco), 2 mM _L_-glutamine, and antibiotics. For the induction of adipocytes differentiation, cells were precultured in basal medium for 2 days and grown to confluence, and then treated with differentiation medium containing 10 μg/ml insulin, 0.25 μM dexamethasone, and 500 μM IBMX (IDI medium) for 2 days, and followed by incubating in basal medium supplemented with insulin alone for 2 days. The cells were further incubated in basal medium for an additional 2 days. At that time, greater than 90% of cells had accumulated multiple lipid droplets and adipocytes differentiation was achieved, which was identified by Oil red O staining. For the experiment, cells were incubated with DMEM + 0.2% BSA at 37 °C for 12 hrs and then were FC-loaded by incubation with full medium containing 10 μg/ml of the ACAT inhibitor 58035 (sigma, USA) plus ox-LDL for indicated times.

### Isolation and oxidation of LDL

Human LDL was isolated and oxidized as described recently by us^34^. In brief, LDL (density = 1.019 − 1.063 g/mL) was isolated from plasma of normolipidemic donors by sequential ultracentrifugation, and incubated with 10 mmol/L CuSO_4_ for 18 h at 37 °C. Following incubation, 0.1 mmol/L ethylenediaminetetraaceticacid (EDTA) was added to prevent further oxidation, and the oxidized LDL was concentrated to 1 mg/mL. The extent of LDL oxidation was assessed based on its increased mobility in an agarose gel (compared with that of native LDL) and also by the presence of increased concentrations of TBARS in the sample. Typically, ox-LDL preparations had TBARS of >30 μmol/g protein and a relative mobility index on agarose gels of 2.0–2.5 when compared with that of native LDL. Lipoproteins were stored at 4 °C in the dark and freshly prepared every two weeks.

### Measurement of adipokine levels in medium

Visfatin and resistin levels in cell medium were detected with the Elisa assay kits (Bluegene, Shanghai, China) according to the manufacturers’ instructions.

### ER membrane fractionation and free cholesterol (FC) assay

Endoplasmic reticulum was prepared as previously described^35^. In detail, a sucrose step gradient was prepared by layering 1.5 ml of 1.1 M sucrose, 1.3 ml of 0.88 M sucrose, and 1.3 ml of 0.58 M sucrose in a 6.8 ml centrifuge tube, followed by refrigeration for 2 h at 4 °C. Cells from three 150 mm plates were scraped carefully, washed with PBS, resuspended into 2 ml of low-ionic strength buffer (10 mM Tris-HCl, pH 7.5, 0.5 mM MgCl_2_, 1× proteinase inhibitor), and incubated on ice for 15 min. The cells were then homogenized with 40 strokes in a Dounce homogenizer with a type A pestle. The homogenates were then made isotonic by the addition of 0.4 ml of a low-ionic strength buffer containing 1.46 M sucrose and then centrifuged at 10,000 × g for 15 min at 4 °C. The supernatant (2.4 ml) was divided into two equal portions of 1.2 ml, loaded onto two sucrose density gradient tubes, and centrifuged at 100,000 × g for 2 h at 4 °C. This procedure resulted in visible bands at each of the four interfaces plus a pellet. Five fractions were collected. The pellet, which was enriched in endoplasmic reticulum, was washed twice and then resuspended in 200 μl of Buffer A (0.25 M sucrose, 0.15 M KCl, 3 mM β-mercaptoethanol, 20 μM CaCl_2_, 10 mM Tris–HCl, pH 7.5). Equal amounts of protein from each fraction were analyzed by SDS–PAGE, and immunoblotting for the ER marker ribophorin1 was performed. Free cholesterol (FC) content of the whole cell lysate and ER-enriched pellet was assayed using a cholesterol assay kit (Biovision, USA) according to the manufacturer’s instruction and normalized by the corresponding protein mass.

### Western Blots

Cellular or nuclear extracts were prepared as previously described^36^. They were then subjected to western blot analyses using CHOP (Santa Cruz), bip (Abcam), phosphorylated PERK (Santa Cruz), phosphorylated eIF2α(Santa Cruz), ATF6 (Santa Cruz), histone H3 (Abcam), SR-BI (Novus Biologicals), PDZK1 (Abcam) and β-actin (Sigma) antibodies. The proteins were visualized and quantified using an enhanced chemiluminescence method (Pierce) and quantified using a Chemiluminescence imaging system (Bioshine Chemi Q 4800mini, Shanghai, China).

### Quantitative real-time PCR

Total cellular RNA was isolated by TRIZOL Reagent (Invitrogen). cDNA synthesis was performed using MuLV Reverse Transcriptase (Applied Biosystems). Real-time PCR was performed using a SYBR green PCR master mix kit (TianGen Biotech). The primers used for real-time PCR were as follows: SR-BI Forward ATCTGGTGGACAAATGGAA; SR-BI Reverse GAAGCGATACGTGGGAAT; PDZK1 Forward TGACGGTGTGGTGGAAATG; PDZK1 Reverse TGGCAGTAAAGAAGTGGAGAG; GAPDH Forward TGACGTGCCGCCTGGAGA AA; GAPDH Reverse AGTGTAGCCCAAGATGCCCTTCAG. The data was analyzed by using Rotor-gene Q software (Qiagen). Relative mRNA levels were calculated by the method of 2^−DDCt^.

### Flow cytometry

Cells was stained with 1 μg/ml SR-BI antibody for 60 min. After washing in PBS for 3 times, the cells were resuspended in FITC-labeled anti-rabbit antibody. After incubating for 30 min, the cells were washed with ice cold PBS for 3 times and then analyzed on Flow Cytometer (BD FACS Calibur).

### Small interfering RNA (siRNA) transfection

Cells were transfected with specific siRNA oligomers (80 nM, Sigma, USA) directed against SR-BI using Lipofectamine 2000 transfection reagent (Invitrogen) according to the manufacturer’s instructions. Scrambled siRNA oligomers were used as a negative control. After transfection for 48 hr, the cells were exposed to ox-LDL. The silencing of target genes was validated by western blot.

### Endotoxin measurement

The contents of endotoxin present in isolated HDL were measured by Dynamic Turbidimetric methods on a bacterial endotoxin autoanalyzer (TDTF, BET-24A, Tianjin, China).

### Statistical Analysis

Statistical analysis was performed by one-way analysis of variance (ANOVA) test and univariate and multivariate logistic regression analyses with the GraphPad Prism programme ver.4.0. Multiple comparison between the groups was performed using Tukey method. Results are expressed as means ± SEM. P values less than 0.05 were considered significant.

## Additional Information

**How to cite this article**: Song, G. *et al.* High-density lipoprotein inhibits ox-LDL-induced adipokine secretion by upregulating SR-BI expression and suppressing ER Stress pathway. *Sci. Rep.* **6**, 30889; doi: 10.1038/srep30889 (2016).

## Supplementary Material

Supplementary Information
