# Supplementary material for: High-density lipoprotein inhibits ox-LDL-induced adipokine secretion by upregulating SR-BI expression and suppressing ER Stress pathway
Source: Sci Rep. 2016 Jul 29;6:30889. doi: 10.1038/srep30889 (PMC4965769; doi:10.1038/srep30889)
Supplement: Supplementary Information [file srep30889-s1.doc]

**High-density lipoprotein inhibits ox-LDL-induced adipokine secretion by upregulating SR-BI expression and suppressing ER Stress pathway**

Guohua Song^§ *1^, Xia Wu^§1,2,4^, Pu Zhang^§4^, Yang Yu^1^, Mingfeng Yang^1^, Peng Jiao^1^, Ni Wang^5^, Haiming Song^5^, You Wu^1^, Xiangjian Zhang^3^, Huaxia Liu^*2^, Shucun Qin^*1^

1 Institute of Atherosclerosis, Key Laboratory of Atherosclerosis in Universities of Shandong, TaiShan Medical University, Taian, China

2 Institute of Nursing, TaiShan Medical University, Taian, China

3 Hebei Collaborative Innovation Center for Cardio-cerebrovascular Disease and Hebei Key Laboratory of Vascular Homeostasis, Shijiazhuang, China, 050000

4 Central Hospital of Taian City, Taian, China

5 Maternal and child health hospital of Daiyue District, Taian, China

Correspondence to ^*^Shucun Qin, Institute of Atherosclerosis, Taishan Medical University, No. 2 Yingsheng East Road, Taian, Shandong 271000, P. R. China, E-mail: [shucunqin@hotmail.com](mailto:shucunqin@hotmail.com), Telephone: 86-538-6237252, Fax: 86-538-6225275. ^*^Huaxia Liu, Institute of Nursing, TaiShan Medical University, Taian, ShanDong, China, E-mail: [huaxia66@163.com](mailto:huaxia66@163.com) , Telephone: 86-538-6238112 , Fax: 86-538-6238121. ^*^Guohua Song, Institute of Atherosclerosis, Taishan Medical University, No. 2 Yingsheng East Road, Taian, Shandong 271000, P. R. China, E-mail: [girl_sapphire@hotmail.com](mailto:girl_sapphire@hotmail.com)

^§^ These authors contributed equally to this work.

Supplemental Table 1. Demographic and Basic Medical Characteristics of the Study Population

| **variable** |  |
| --- | --- |
| n | 34 |
| Age (years) | 62.4 ± 4.8 |
| Gender-Male | 22/34 |
| Gender-Female | 12/34 |
| Mean Systolic BP (mmHg) | 143.1 ± 21.9 |
| Mean Diastolic BP (mmHg) | 87.4 ± 12.5 |
| Height (cm) | 164.5 ± 13.5 |
| Weight (kg) | 73.8 ± 10.8 |
| Waist Circumference (cm) | 98.9 ± 7.9 |
| BMI (kg/m^2^) | 27.3 ± 3.8 |
| Triglycerides (mmol/L) | 2.81 ± 1.08 |
| HDL cholesterol (mmol/L) | 1.14 ± 0.28 |
| LDL cholesterol (mmol/L) | 3.22 ± 0.73 |
| Fasting glucose (mmol/L) | 7.45 ± 3.12 |

Supplemental Table 2. The contents of endotoxin in HDL particles

| **Sample number** | **Endotoxin concentration (EU/ml)** | **HDL protein concentration (μg/ml)** | **Endotoxin content (EU/100μg protein)** | **Mean±SD**  **(EU/100μg protein)** |
| --- | --- | --- | --- | --- |
| **1** | 0.01 | 100 | 0.01 | 0.023 ± 0.015 |
| **2** | 0.11 | 1000 | 0.011 |
| **3** | 0.9 | 3000 | 0.03 |
| **4** | 0.04 | 100 | 0.04 |

EU, endotoxin units
